# Outpatient antibiotic prescribing in the United States: 2000 to 2010

**DOI:** 10.1186/1741-7015-12-96

**Published:** 2014-06-11

**Authors:** Grace C Lee, Kelly R Reveles, Russell T Attridge, Kenneth A Lawson, Ishak A Mansi, James S Lewis, Christopher R Frei

**Affiliations:** 1 College of Pharmacy, The University of Texas at Austin, Austin, TX, USA; 2 School of Medicine, The University of Texas Health Science Center at San Antonio, Pharmacotherapy Education and Research Center, San Antonio, TX, USA; 3 Feik School of Pharmacy, University of the Incarnate Word, San Antonio, TX, USA; 4 Department of General Internal Medicine, Veterans Affairs North Texas Health Care System and University of Texas Southwestern Medical Center, Dallas, TX, USA

**Keywords:** Antibiotic, Prescribing, Ambulatory care, Antibiotic resistance, Surveillance

## Abstract

**Background:**

The use of antibiotics is the single most important driver in antibiotic resistance. Nevertheless, antibiotic overuse remains common. Decline in antibiotic prescribing in the United States coincided with the launch of national educational campaigns in the 1990s and other interventions, including the introduction of routine infant immunizations with the pneumococcal conjugate vaccine (PCV-7); however, it is unknown if these trends have been sustained through recent measurements.

**Methods:**

We performed an analysis of nationally representative data from the Medical Expenditure Panel Surveys from 2000 to 2010. Trends in population-based prescribing were examined for overall antibiotics, broad-spectrum antibiotics, antibiotics for acute respiratory tract infections (ARTIs) and antibiotics prescribed during ARTI visits. Rates were reported for three age groups: children and adolescents (<18 years), adults (18 to 64 years), and older adults (≥65 years).

**Results:**

An estimated 1.4 billion antibiotics were dispensed over the study period. Overall antibiotic prescribing decreased 18% (risk ratio (RR) 0.82, 95% confidence interval (95% CI) 0.72 to 0.94) among children and adolescents, remained unchanged for adults, and increased 30% (1.30, 1.14 to 1.49) among older adults. Rates of broad-spectrum antibiotic prescriptions doubled from 2000 to 2010 (2.11, 1.81 to 2.47). Proportions of broad-spectrum antibiotic prescribing increased across all age groups: 79% (1.79, 1.52 to 2.11) for children and adolescents, 143% (2.43, 2.07 to 2.86) for adults and 68% (1.68, 1.45 to 1.94) for older adults. ARTI antibiotic prescribing decreased 57% (0.43, 0.35 to 0.52) among children and adolescents and 38% (0.62, 0.48 to 0.80) among adults; however, it remained unchanged among older adults. While the number of ARTI visits declined by 19%, patients with ARTI visits were more likely to receive an antibiotic (73% versus 64%; *P* <0.001) in 2010 than in 2000.

**Conclusions:**

Antibiotic use has decreased among children and adolescents, but has increased for older adults. Broad-spectrum antibiotic prescribing continues to be on the rise. Public policy initiatives to promote the judicious use of antibiotics should continue and programs targeting older adults should be developed.

## Background

Antibiotic resistance has been called one of the world’s most pressing public health problems [1]. Infections due to antibiotic-resistant organisms are associated with loss of productivity, poorer health outcomes and greater health care costs [2-4]. Antibiotic resistance is estimated to cost $60 billion annually in the United States (US) [5].

Antibiotics are one of the most commonly prescribed drugs in the US. The use of antibiotics is the main driver in creating selective pressure for the emergence of antibiotic-resistant bacteria. Nevertheless, antibiotic overuse remains common [6-8]. Despite a high probability of viral etiology, acute respiratory tract infections (ARTIs), such as bronchitis, pharyngitis and sinusitis, account for 75% of all antibiotics prescribed by office-based providers [9-11]. Prior studies have demonstrated that nearly 50% of outpatient antibiotics prescribed are unnecessary [12-15].

To target this growing concern, in 1995, the US Centers for Disease Control and Prevention (CDC) launched its ‘Get Smart: Know When Antibiotics Work’ campaign, a broad-scale, national educational campaign to promote appropriate use of antibiotics, particularly for ARTIs [16]. Subsequently, various reports in the late 1990s and early 2000s demonstrated a decline in antibiotic use [17-19]. Other interventions not directly targeting antibiotic use may have also reduced antibiotic utilization. For example, after the initiation of the routine infant pneumococcal vaccination with the pneumococcal conjugate vaccine (PCV-7) in 2000, reports have shown that the rates of invasive disease due to penicillin-resistant *Streptococcus pneumoniae* have declined. However, it is unknown if these national antibiotic prescribing patterns have continued in more recent years.

The purpose of this study was to describe national trends in outpatient antibiotic prescribing, broad-spectrum antibiotic prescribing, and antibiotic prescribing for ARTIs from 2000 to 2010. We also aimed to examine national trends in ARTI visits and antibiotic prescribing trends during those visits.

## Methods

### Data source

This study utilized data from the Medical Expenditure Panel Survey (MEPS) administered by the Agency for Healthcare Research and Quality [20]. The MEPS are a national set of surveys of individuals, families and their medical providers and employers. It provides national estimates of health care use, expenditures, payment sources and health insurance coverage for the US civilian, non-institutionalized population, annually. The MEPS are conducted using an overlapping panel design, in which a new panel of approximately 35,000 respondents is added each year. Data are collected in five rounds of interviews over two and a half calendar years. Each of the five interviews includes all health care utilization events for a specific time, and these time periods cumulatively covered a two-year period. At the time of the draft of this manuscript, MEPS survey data were complete to year 2010.

The MEPS include three main components: the Household Component (HC), the Medical Provider Component (MPC) and the Insurance Component. The MEPS HC contains information on families and individuals regarding their demographics, health conditions, health status and expenditures. The MEPS MPC includes information provided by a sample of medical providers that supplements the information acquired during completion of the household interviews. Information in the MEPS MPC includes dates of visits, diagnosis and procedure codes, charges and payments. The Pharmacy Component, a sub-component of the MEPS HC, contains the ‘Prescribed Medicines’ files. Medications are obtained from survey respondents; additional details are imputed from data that are obtained from the patients’ pharmacies (for example, National Drug Codes, medication name, date filled and payment sources). Each record in the file represents a unique prescribed medicine event and purchase by the household respondent or member. Expenditures, medical conditions and household characteristics related to the prescribed medicine are also provided.

### Study design and definitions

We performed a retrospective analysis of outpatient antibiotic prescribing from 2000 to 2010. In the MEPS, survey respondents are asked to report health conditions which are then coded by professional coders using the *International Classification of Diseases, 9th Revision, Clinical Modification* (ICD-9-CM) diagnoses codes. These conditions are recorded at the person-level in conjunction with medical events, including outpatient visits and antibiotic purchases. We assessed antibiotic prescribing trends in overall antibiotic use, broad-spectrum antibiotics, outpatient visits, and for common ARTIs that infrequently necessitate an antibiotic: acute nasopharyngitis and upper respiratory tract infection (ICD-9-CM diagnosis codes 460 and 465), bronchitis (466 and 490), influenza (480, 487 and 488), pharyngitis (034, 462 and 463), and sinusitis (461 and 473) [18,21,22]. Outpatient visits included visits with medical providers in office-based settings and clinics, hospital outpatient departments and phone contacts with office-based medical providers.

Therapeutic drug classes were identified by using the National Drug Code directory, generic names, and the Multum Lexicon therapeutic classification database (Cerner Multum, Inc). ‘Broad-spectrum’ antibiotics were defined using the National Committee for Quality Assurance’s (NCQA) ‘Antibiotics of Concern List,’ which includes azithromycin, clarithromycin, fluoroquinolones, amoxicillin-clavulanate, and second- and third-generation cephalosporins [23]. ‘Major antibiotic classes’ included macrolides, cephalosporins, penicillins and fluoroquinolones. Antibiotics for ARTIs were defined as events of antibiotic purchase linked to ICD-9-CM codes mentioned above. We excluded any antibiotic that was reported as a topical route of administration.

### Data and statistical analyses

Antibiotic prescriptions were analyzed at the level of a unique prescribed medicine event and prescription purchase. Prescription rates were defined as the annual number of antibiotic prescriptions divided by the overall US civilian non-institutionalized population. Population denominators were derived from the MEPS HC. The MEPS estimate US populations based on sampled persons in the target population (civilian non-institutionalized) for the entire year. Beginning in 2001, MEPS transitioned to 2000 census-based population estimates for post-stratification and ranking; prior to 2001, 1990 census-based estimates were used. The sample design of the survey includes stratification, clustering, multiple stages of selection and disproportionate sampling. Furthermore, the MEPS use sampling weights to reflect adjustments for survey nonresponse and adjustments to population control totals from the Current Population Survey [20]. Next, we determined if changes in antibiotic use were uniform across three age groups: children and adolescents (<18 years), adults (18 to 64 years), and older adults (≥65 years). To account for the aging population, groups were further stratified into a total of six subgroups: <5 years, 5 to 17 years, 18 to 49 years, 50 to 64 years, 65 to 79 years, and ≥80 years.

We conducted analyses evaluating trends in both ARTI visits and visit-based prescribing. ARTI outpatient visit rates were defined as the annual number of ARTI outpatient visits divided by the corresponding population estimate. Rates in the first study year (2000) were compared with the final study year (2010) and reported per 1,000 persons per year. Risk ratios and 95% confidence intervals (95% CIs) were calculated.

ARTI visit-based prescribing was determined as described in the study design and definitions. The only difference was that the data were limited to antibiotic events that occurred during an ARTI visit. Proportions were estimated accounting for the survey design and tested using the design-based *F* test. All analyses were adjusted to account for the MEPS complex study design using weights, clustering and stratification to derive national estimates. SPSS 20.0® (IBM Corp, Armonk, NY, USA) was used for all statistical analyses. *P*-values of <0.05 indicated statistical significance.

## Results

### Antibiotic prescription rates

**Figure 1:**
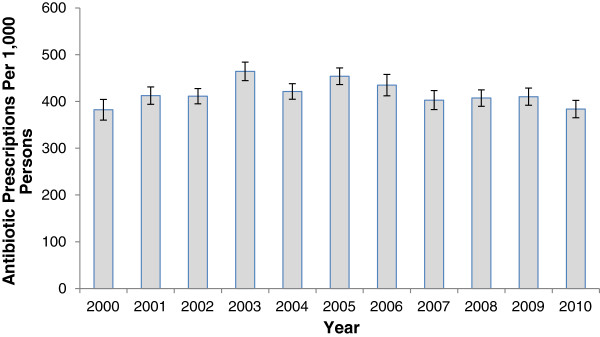
**Antibiotic prescribing in the United States, 2000 to 2010.** Data are from the Medical Expenditure Panel Surveys. Error bars indicate 95% confidence intervals. Rates are per 1,000 population.

**Figure 2:**
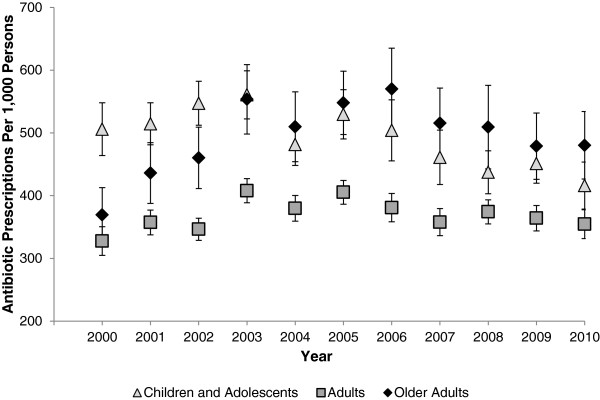
**Age-based antibiotic prescribing in the United States, 2000 to 2010.** Data are from the Medical Expenditure Panel Surveys. Error bars indicate 95% confidence intervals. Rates are per 1,000 population.

During the study period (2000 to 2010), an estimated 1.4 billion outpatient antibiotics were dispensed in the US. The annual number of outpatient antibiotic prescriptions purchased ranged from 106 million in 2000 to 134 million in 2003.Annual antibiotic prescription rates are shown in Figure 1. Antibiotic prescription rates remained relatively stable over the study period from 382 (95% CI 360 to 404) per 1,000 persons in 2000 to 384 (365 to 403) per 1,000 persons in 2010. Age-based antibiotic prescribing rates are shown in Figure 2. Among children and adolescents, the annual rate of antibiotic prescriptions remained stable from 2000 to 2003; subsequently, rates declined by 25% from 2003 to 2010. For adults, antibiotic prescribing peaked in 2003 and then remained stable until 2010. In contrast, rates for older adults increased 30% from 2000 to 2010.

Table 1 provides a comparison of antibiotic prescribing between 2000 and 2010. Prescription rates in children and adolescents decreased 18% from 2000 to 2010 (risk ratio (RR) 0.82, 95% CI 0.72 to 0.94), mainly driven by children <5 years of age (0.72, 0.65 to 0.81). Among adults (18 to 64 years of age), rates were similar in 2000 and 2010 (1.08, 0.93 to 1.26). In contrast, rates increased significantly for older adults (>65 years of age) from 2000 to 2010 (1.30, 1.14 to 1.49). The most substantial increase was seen among those ≥80 years old, where antibiotic prescription rates doubled from 2000 to 2010 (1.96, 1.69 to 2.28).

**Table 1:** Antibiotic prescribing in the United States, 2000 and 2010

**Antibiotic prescription rates (per 1,000 persons)**^**a**^	**2000**	**2010**	**Risk ratio (95%CI)**
**Overall antibiotics**			
United States population	379	386	1.02 (0.88 to 1.17)
Children and adolescents (<18)	506	416	0.82 (0.72 to 0.94)
0 to 4	776	562	0.72 (0.65 to 0.81)
5 to 17	405	358	0.88 (0.77 to 1.02)
Adults (18 to 64)	328	354	1.08 (0.93 to 1.26)
18 to 49	317	329	1.04 (0.89 to 1.21)
50 to 64	368	410	1.11 (0.97 to 1.28)
Older adults (≥65)	369	480	1.30 (1.14 to 1.49)
65 to 79	405	473	1.17 (1.02 to 1.33)
≥80	254	498	1.96 (1.69 to 2.28)
**Broad-spectrum antibiotics**^**b**^			
United States population	223	471	2.11 (1.81 to 2.47)
Children and adolescents (<18)	223	400	1.79 (1.52 to 2.11)
0 to 4	268	400	1.49 (1.28 to 1.74)
5 to 17	191	400	2.09 (1.76 to 2.49)
Adults (18 to 64)	205	499	2.43 (2.07 to 2.86)
18 to 49	194	499	2.57 (2.18 to 3.04)
50 to 64	237	499	2.11 (1.80 to 2.46)
Older adults (≥65)	287	482	1.68 (1.45 to 1.94)
65 to 79	293	489	1.67 (1.44 to 1.93)
≥80	260	466	1.79 (1.54 to 2.09)
**Antibiotics for ARTIs**^**c**^			
United States population	175	102	0.58 (0.46 to 0.74)
Children and adolescents (<18)	326	139	0.43 (0.35 to 0.52)
0 to 4	268	138	0.51 (0.41 to 0.63)
5 to 17	352	139	0.40 (0.33 to 0.48)
Adults (18 to 64)	151	94	0.62 (0.48 to 0.80)
18 to 49	171	97	0.57 (0.44 to 0.73)
50 to 64	104	87	0.83 (0.63 to 1.11)
Older adults (≥65)	74	69	0.94 (0.67 to 1.30)
65 to 79	79	80	1.01 (0.74 to 1.37)
≥80	58	41	0.72 (0.58 to 1.07)

^a^MEPS population estimates in 2000: 36,053,864 (0 to 17 years of age); 106,057,609 (0 to 64 years of age); 29,706,554 (>65 years and older). MEPS population estimates in 2010: 74,834,192 (0 to 17 years of age); 192,582,188 (0 to 64 years of age); 41,157,595 (>65 years and older); ^b^broad-spectrum antibiotics are defined per the National Committee for Quality Assurance which includes azithromycin, clarithromycin, fluoroquinolones, amoxicillin-clavulanate, and second- and third-generation cephalosporins; ^c^ARTIs include the following ICD-9-CM codes: bronchitis, 466 and 490; pharyngitis, 034, 462 and 463; sinusitis, 461 and 473; acute nasopharyngitis and upper respiratory tract infection, 460 and 465; and influenza, 480, 487 and 488. ARTIs, acute respiratory tract infections; MEPS, Medical Expenditure Panel Surveys; 95% CI, 95% confidence interval.

### Broad-spectrum antibiotic prescription rates

Table 1 compares broad-spectrum antibiotic prescription rates in 2000 versus 2010. Rates of broad-spectrum antibiotic prescriptions doubled from 2000 to 2010 (2.11, 1.81 to 2.47). Among children and adolescents, broad-spectrum antibiotic prescriptions increased 49% in those younger than 5 years of age (1.49, 1.28 to 1.74) and doubled among those 5 to 17 years of age (2.09, 1.76 to 2.49). The adult population experienced the greatest increase in broad-spectrum antibiotic prescribing (2.43, 2.18 to 3.04), including a 2.5-fold increase among those 18 to 49 years of age (2.57, 2.18 to 3.04) and a two-fold increase in those 50 to 64 years of age (2.11, 1.80 to 2.46). Among older adults, broad-spectrum antibiotic prescribing rates increased 68% from 2000 to 2010 (1.68, 1.45 to 1.94).

### Antibiotic prescription rates for ARTIs

Table 1 also depicts antibiotic prescription rates for ARTIs. The US population experienced a significant decrease in ARTI antibiotic prescriptions from 2000 to 2010 (0.58, 0.46 to 0.74); however, this decrease occurred only among groups younger than 50 years of age: 49% decrease among children ≤4 years of age (0.51, 0.41 to 0.63), 60% decrease among those 5 to 17 years of age (0.40, 0.33 to 0.48), and 43% decrease among those 18 to 49 years of age (0.57, 0.44 to 0.73). In contrast, there were no significant changes among groups ≥50 years of age.

### Broad-spectrum antibiotic prescription rates for ARTIs

Broad-spectrum antibiotic prescriptions for ARTIs in 2000 versus 2010 are compared in Table 2. In the US population, broad-spectrum antibiotic prescriptions more than doubled from 2000 to 2010 (2.34, 2.03 to 2.68). This increase was observed for all age groups, including children and adolescents (1.81, 1.56 to 2.09), adults (2.77, 2.42 to 3.17), and older adults (2.25, 2.02 to 2.52).

**Table 2:** Broad-spectrum prescribing for ARTIs, 2000 and 2010

**Antibiotic prescription rates (per 1,000 antibiotic prescriptions)**^**a,b**^	**2000**	**2010**	**Risk ratio (95% CI)**
United States population	289	675	2.34 (2.03 to 2.68)
Children and adolescents (<18)	273	436	1.81 (1.56 to 2.09)
0 to 4	314	466	1.48 (1.28 to 1.71)
5 to 17	237	516	2.18 (1.87 to 2.54)
Adults (18 to 64)	287	796	2.77 (2.42 to 3.17)
18 to 49	274	766	2.80 (2.45 to 3.21)
50 to 64	339	871	2.57 (2.27 to 2.91)
Older adults (≥65)	457	1030	2.25 (2.02 to 2.52)
65 to 79	464	950	2.05 (1.83 to 2.28)
≥80	423	1416	3.35 (3.00 to 3.73)

^a^Broad-spectrum antibiotics are defined per the National Committee for Quality Assurance which includes azithromycin, clarithromycin, fluoroquinolones, amoxicillin-clavulanate, and second- and third-generation cephalosporins; ^b^ARTI visits were outpatient visits with one or more of the following ICD-9-CM codes: bronchitis, 466 and 490; pharyngitis, 034, 462 and 463; sinusitis, 461 and 473; acute nasopharyngitis and upper respiratory tract infection, 460 and 465; and influenza, 480, 487 and 488. ARTIs, acute respiratory tract infections; 95% CI, 95% confidence interval.

### Prescription rates for major antibiotic classes

Penicillin prescribing decreased for children and adolescents (0.80, 0.69 to 0.92), adults (0.83, 0.78 to 0.94) and older adults (0.80, 0.66 to 0.98). Cephalosporin prescribing remained stable for children and adolescents (0.90, 0.71 to 1.15), but decreased for adults (0.73, 0.56 to 0.94) and older adults (0.78, 0.61 to 0.99). In contrast, macrolide prescribing significantly increased for all age groups: children and adolescents (1.77, 1.43 to 2.17), adults (1.33, 1.12 to 1.57) and older adults (1.40, 1.16 to 1.69). Finally, fluoroquinolone prescribing remained stable in adults (0.99, 0.76 to 1.30) and older adults (0.87, 0.72 to 1.05). There were too few events for fluoroquinolones to estimate use among children and adolescents.

### ARTI visits

From 2000 to 2010, the survey estimated 3.1 billion office-based provider visits and hospital outpatient provider visits. The rate of outpatient visits did not change significantly from 2000 to 2010 for any of the age groups. In 2000, ARTI visits accounted for 28% of all outpatient visits; in 2010, ARTI visits accounted for approximately 21% of all outpatient visits.

Table 3 compares ARTI visit rates in 2000 and 2010. The national ARTI visit rates decreased 19% from 2000 to 2010 (0.81, 0.67 to 0.98). This was mainly driven by a 33% decrease among children younger than 5 years of age (0.67, 0.58 to 0.77) and a 32% decrease among adults 50 to 64 years of age (0.68, 0.54 to 0.85). ARTI visit rates were not different in older adults between 2000 and 2010 (0.83, 0.65 to 1.07).

**Table 3:** ARTI visits in the United States, 2000 and 2010

**Visit rates (per 1,000 persons)**^**a**^	**2000**	**2010**	**Risk ratio (95% CI)**
United States population	239	193	0.81 (0.67 to 0.98)
Children and adolescents (<18)	359	302	0.84 (0.72 to 0.97)
0 to 4	481	323	0.67 (0.58 to 0.77)
5 to 17	296	292	0.99 (0.84 to 1.16)
Adults (18 to 64)	207	172	0.83 (0.68 to 1.02)
18 to 49	216	198	0.92 (0.76 to 1.11)
50 to 64	185	125	0.68 (0.54 to 0.85)
Older adults (≥65)	137	114	0.83 (0.65 to 1.07)
65 to 79	148	126	0.85 (0.67 to 1.08)
≥80	104	79	0.76 (0.57 to 1.02)

^a^ARTI visits were outpatient visits with one or more of the following ICD-9-CM codes: bronchitis, 466 and 490; pharyngitis, 034, 462, and 463; sinusitis, 461 and 473; acute nasopharyngitis and upper respiratory tract infection, 460 and 465; and influenza, 480, 487, and 488. ARTI, acute respiratory tract infection; 95% CI, 95% confidence interval.

### Antibiotic prescriptions during ARTI visits

Although ARTI visits decreased over the study period, the proportion of antibiotic prescriptions associated with provider visits for ARTIs increased significantly from 2000 to 2010 (64% versus 73%, *P* <0.0001). Significant increases were observed in children and adolescents (68% versus 75%; *P* = 0.001) and adults (62% versus 74%; *P* <0.0001). Rates were similar among older adults (61% versus 66%; *P* = 0.3).

### Broad-spectrum antibiotic prescriptions during ARTI visits

**Figure 3:**
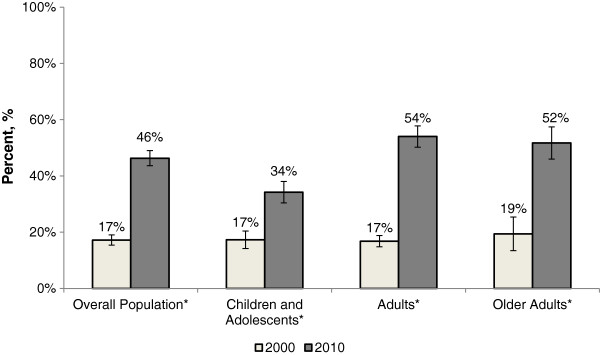
**Broad-spectrum antibiotic prescribing during acute respiratory tract infection visits in the United States, 2000 to 2010.** *P <0.0001. Acute respiratory tract infection includes acute nasopharyngitis, upper respiratory tract infection, bronchitis, influenza, pharyngitis and sinusitis. Data are from the Medical Expenditure Panel Surveys. Error bars represent 95% confidence intervals. Rates are per 1,000 population.

There was also a substantial increase in broad-spectrum antibiotic prescribing during ARTI visits (Figure 3). In 2000, broad-spectrum antibiotics comprised 17% of total antibiotics prescribed in outpatient ARTI visits. By 2010, broad-spectrum antibiotics comprised 46% (*P* <0.001) of all antibiotics prescribed for ARTIs. This significant increase was consistent across all age groups: children and adolescents (17% versus 34%, *P* <0.0001), adults (17% versus 54%, *P* <0.0001) and older adults (19% versus 52%, *P* <0.0001).

## Discussion

This study describes antibiotic prescribing in the US from 2000 to 2010. Overall, antibiotic use has remained stable; however, there were opposing trends among different age groups. While antibiotic use decreased among children and adolescents, there was no change among adults, and older adults experienced an increase.

Earlier studies have documented declining antibiotic prescription rates among children and adolescents [18,21,24]. McCaig *et al*. demonstrated a 40% decrease in antibiotic prescribing from 1989–1990 through 1999–2000 among children <15 years of age. In a subsequent study, Finkelstein *et al*. observed a 24% decrease in antibiotic prescribing from 1996 to 2000 among children within nine US health plans [25]. Our study demonstrates that this decline in antibiotic use among children has continued through to 2010, representing a major success in the US. These findings likely reflect a combination of several factors. This includes national, state and local initiatives aimed at reducing misdiagnoses of ARTIs and decreasing inappropriate prescribing of antibiotics. Also, the introduction of routine infant vaccinations with the PCV-7 in 2000 coincided with reduced incidence of invasive pneumococcal disease and otitis media, which can decrease antibiotic use [26,27]. In addition, since 2008, influenza vaccinations became recommended for all children six months and older which may have had a positive effect on the occurrence of ARTIs [28].

Other age groups did not experience this decline. In the beginning of the study period, children and adolescents had the highest rate of antibiotic prescriptions compared to adults and older adults. However, older adults surpassed children and adolescents to attain the highest antibiotic prescribing rate in 2004; this was sustained through the end of the study period. A prior study demonstrated that, while overall antibiotic prescriptions remained stable among persons ≥5 years of age, there was a significant increase in antibiotic use among persons ≥50 years of age from 1995 to 2006 [24]. Recently, Zhang *et al*. estimated that the rate of outpatient antibiotics among Medicare Part D beneficiaries (>65 years of age) in 2007 to 2009 was 1.10 person/year [29]; this rate was higher than the antibiotic prescription rate among those <65 years of age (0.88 person/year) [30]. Our study validates and expands on these findings by demonstrating an increase in antibiotic use over the last decade among US older adults (370/1,000 persons in 2000 to 480/1,000 persons in 2010).

Perhaps the most alarming finding of this study is the substantial increase of broad-spectrum antibiotic prescribing. Previously, a cross-sectional study identified an increase in broad-spectrum antibiotic prescribing from 24% to 48% among adults and from 23% to 40% among children from 1991–1992 to 1998–1999 [22]. Moreover, a national study demonstrated that azithromycin and fluoroquinolone use increased nine-fold and five-fold, respectively, from 1995–1996 to 2005–2006 [21]. Our study suggests that this upsurge of broad-spectrum antibiotic use is at an unprecedented high.

Changes in antibiotic prescribing rates might have resulted from changes in provider prescribing behavior and/or frequency of outpatient visits or encounters. Changes in outpatient visit rates for ARTIs might reflect changes in factors such as public behavior from education, insurance status, disease severity, and internet-based advice and prescribing [24]. Therefore, we conducted analyses evaluating trends in both ARTI visits and visit-based prescribing. Our study found that ARTI visit rates have decreased in the last decade; however, when patients experience an outpatient ARTI visit, they are more likely to receive an antibiotic. Steinman *et al*. previously demonstrated a decline in visit-related antibiotic prescribing rates between 1991–1992 and 1998–1999 for various respiratory tract infections [22]. Similarly, McCaig *et al*. found decreasing trends in visit-based ARTI antibiotic prescribing among children and adolescents from 1989 to 2000 [18]. This decrease was sustained in a subsequent study that evaluated antibiotic use among children ≤5 years of age, from 1995–1996 to 2005–2006 [25]. However, longitudinal evaluation of these studies suggests that most of the decrease occurred before 2000 [25,31]. When comparing antibiotic prescribing during ARTI visits, our data indicate a notable 9% increase from 2000 to 2010.

Taking into account reduced ARTI visit-rates, these trends suggest that, although patients may have been less likely to make a provider visit, clinicians had a higher propensity to treat these conditions with an antibiotic in 2010. Moreover, when antibiotics were prescribed during an ARTI visit, broad-spectrum agents were increasingly being selected. In 2010, broad-spectrum antibiotics comprised almost half of all antibiotics prescribed in US outpatient provider visits. Overall, this translates into nearly 32 million antibiotic prescriptions for respiratory tract infections, comprised largely of broad-spectrum agents.

Finally, our study suggests that older adults experienced a significant increase in antibiotic prescribing and constituted the greatest proportion of patients receiving broad-spectrum antibiotics. This highlights the need for further studies to better understand antibiotic use among older adults. The older adult population is rapidly growing compared to other US age groups. As the ‘baby boomer’ generation approaches retirement age, they will comprise the largest US health care sector. Targeted quality initiatives for this population are needed. Currently, the NCQA has initiated quality improvement measures for antibiotic prescribing for children and adults; however, no such metrics exist for those 65 years of age and older.

This study has limitations. First, the MEPS database does not provide information related to patient signs and symptoms, medication allergies, microbial etiology or severity of the condition; therefore, direct assessment of appropriate antibiotic prescribing is difficult. Because we relied on ICD-9-CM codes, diagnoses are subject to misclassification. Second, we were not able to distinguish between first and follow-up visits. The inclusion of follow-up visits may have resulted in underestimation of visit-based antibiotic prescriptions. Antibiotic data were based on antibiotic prescription purchases; thus, actual antibiotic prescribing might have been underestimated and may not necessarily reflect actual antibiotic consumption. Third, while this study was based on national population estimates, wide confidence intervals seen in point estimates of specific age groups might have been associated with smaller sample sizes, thereby resulting in possible unstable estimates of standard error. Fourth, while this study focused on ARTIs, varying trends for different indications that might affect total antibiotic use were not directly evaluated in this study. Lastly, the MEPS do not include federal institutions (that is, federal, military and Veterans Affairs health care facilities), thus our estimates may not be generalizable to those populations.

## Conclusions

In conclusion, overall antibiotic use has decreased among children and adolescents, but has increased among older adults. Broad-spectrum antibiotic prescribing has increased among all age ranges. ARTI visits have declined; however, patients who present for ARTI visits are more likely to receive an antibiotic than in the past. Our study highlights the persistent problem of antibiotic overuse. Public policy initiatives to promote the judicious use of antibiotics should continue and programs targeting older adults should be developed.

## Competing interests

CRF has received research grants and/or served as a scientific consultant/advisor for the NIH, AstraZeneca, Bristol-Myers Squibb, Elan, Forest, Ortho McNeil Janssen Pharmaceuticals, and Pfizer. GCL, KRR, RTA, KAL, IAM and JSL declare that they have no competing interests.

## Authors’ contributions

All authors had full access to the data, which contain publicly accessible data sets available from the Agency of Health Care Research and Quality. Study concept: GCL and CRF; acquisition of data: GCL; analysis and interpretation of data: GCL, KAL, CRF; drafting of the manuscript: GCL; critical revisions of the manuscript for important intellectual content: GCL, KRR, RTA, KAL, IAM, JSL and CRF; statistical expertise: GCL, KAL, CRF; administrative, technical, or material support: GCL; study supervision: CRF. All authors read and approved the final manuscript.

## Pre-publication history

The pre-publication history for this paper can be accessed here:

http://www.biomedcentral.com/1741-7015/12/96/prepub
